# CAl_4_X_4_ (X = Te, Po): Double Aromatic Molecular Stars Containing Planar Tetracoordinate Carbon Atoms

**DOI:** 10.3390/molecules28073280

**Published:** 2023-04-06

**Authors:** Li-Xia Bai, Jin-Chang Guo

**Affiliations:** Nanocluster Laboratory, Institute of Molecular Science, Shanxi University, Taiyuan 030006, China

**Keywords:** planar tetracoordinate carbon, global minimum, chemical bonding, double aromaticity, stability

## Abstract

Planar tetracoordinate carbon (ptC) species are scarce and exotic. Introducing four peripheral Te/Po auxiliary atoms is an effective strategy to flatten the tetrahedral structure of CAl_4_ (*T_d_*, ^1^A_1_). Neutral CAl_4_X_4_ (X = Te, Po) clusters possess quadrangular star structures containing perfect ptC centers. Unbiased density functional theory (DFT) searches and high-level CCSD(T) calculations suggest that these ptC species are the global minima on the potential energy surfaces. Bonding analyses indicate that 40 valence-electron (VE) is ideal for the ptC CAl_4_X_4_ (X = Te, Po): one delocalized π and three σ bonds for the CAl_4_ core; four lone pairs (LPs) of four X atoms, eight localized Al–X σ bonds, and four delocalized Al–X–Al π bonds for the periphery. Thus, the ptC CAl_4_X_4_ (X = Te, Po) clusters possess the stable eight electron structures and 2π + 6σ double aromaticity. Born–Oppenheimer molecular dynamics (BOMD) simulations indicate that neutral ptC CAl_4_X_4_ (X = Te, Po) clusters are robust.

## 1. Introduction

The concept of tetrahedral carbon (thC) was proposed by van’t Hoff [1] and Le Bel [2] in 1874 and it had dominated chemistry for nearly 150 years. Planar tetracoordinate carbon (ptC) was first put forward in a hypothetical transition-state structure by Monkhorst in 1968 [3] although he did not try to stabilize such ptC species. It may be motivated by pure chemical curiosity to understand and design non-classical chemical bonds surrounding a carbon center. Tetrahedral or planar? The ptC species are usually unstable in energies with respect to their thC structures. How do we stabilize the ptC species? In response to this question, Hoffmann and coworkers proposed the effective “electronic” strategy in 1970, based on bonding analysis of hypothetical planar CH_4_ [4]. Ligands are crucial for stabilizing the ptC species, which need to be the σ-donors and π-acceptors. The first stable ptC local minimum, 1,1-dilithiocyclopropane, was theoretically designed by Schleyer and coworkers [5]. Although unrecognized at the time, the first experimental example of a ptC compound was reported in the X-ray structure of a vanadium 2,6-dimethoxyphenyl complex by Cotton et al. in 1977 [6]. Since then, a variety of planar tetra-, penta-, and hexa-coordinate carbon (ptC, ppC, and phC) novel clusters, molecules, complexes, and even nanomaterials have been reported [7,8,9,10,11,12,13,14].

The neutral CAl_4_ possesses the typical tetrahedral *T_d_* structure, in which there is no effective bonding between the adjacent Al ligands. How do we flatten it? It is important to strengthen the bonding between the adjacent Al ligands. One way of doing so is adding one or two electrons to the bonding orbitals between the ligands. Using photoelectron spectroscopy (PES) combined with theoretical calculation, Wang and Boldyrev identified the ptC structures of C${\mathrm{Al}}_{4}^{-}$, NaAl_4_C^−^, CAl_3_Si^−^, and CAl_3_Ge^−^ in 1999–2000 [15,16,17]. These ptC species can be seen as the derivatives of the 18-electron C${\mathrm{Al}}_{4}^{2-}$ cluster (except for C${\mathrm{Al}}_{4}^{-}$ which is iso-electronic with the previously reported CSi_2_Al_2_ cluster) [18]. In these ptC species, the Al ligands not only act as σ electron donors but also as π electron acceptors. Another way to flatten the CAl_4_ cluster is by introducing one or more auxiliary atoms (or ions) at the Al-Al edges. The auxiliary atoms can strengthen the interaction between the ligands. The auxiliary atoms also help disperse the electrons over a larger area, therefore reducing the electrostatic repulsion of bonding electrons between the ligands and the carbon center. For example, ptC CAl_4_Al^−^ and CAl_4_H^−^, which have been experimentally identified [19,20]. Recently, star-like ppB BAl_5_$S_{5}^{+}$, ppC CAl_5_$O_{5}^{+}$, and CB_5_$S_{5}^{+}$ were predicted as the ground state structures [21,22,23]. The star-like σ-aromatic MAl_6_S_6_ (M = Ni, Pd, Pt) species containing the peculiar planar hexacoordinate transition metals were reported by us [24]. 

Can we use oxygen-group X (X = O, S, Se, Te, Po) atoms to flatten CAl_4_ and design CAl_4_-based molecular stars? The answer seems to be yes. In the design of ptC clusters, geometric size match is intuitively crucial. Of equal importance is the number of electrons in the system. The perfect match between the cavity of the Al_4_ ring and the volume of the central ptC atom is crucial. Whether the cavity of the Al_4_ ring is too large or too small, it is difficult to stabilize the central ptC atom. By tuning the number and type of auxiliary atoms, after a lot of attempts, we eventually found that the Al_4_X_4_ (X = Te, Po) ring is suitable to hold the ptC center in geometry. It should be noted that *D*_4*h*_ Al_4_X_4_ (X = O, S, Se,) are only the transition states on the potential energy surfaces at the B2PLYP-D3(BJ)/def2-TZVPP level. These imaginary frequencies correspond to the A_2u_ vibrational mode, in which the central atom C moves up and down along the fourfold axis. Distortion of the *D*_4*h*_ structure in the A_2u_ mode leads to the *C*_4*v*_ CAl_4_X_4_ (X = O, S, Se) minima, in which the C atoms lie about 0.96, 0.61, and 0.47 Å above the Al_4_ plane, respectively. Thus, only *D*_4*h*_ CAl_4_X_4_ (X = Te, Po) are perfect ptC minima.

To predict experimentally observable ptC species under annealing conditions, it is best that the target structure has the lowest energy. Excitingly, further calculations suggest that the neutral ptC CAl_4_X_4_ (X = Te, Po) are the global minima (GMs) on the potential energy surfaces. The abbreviation “GM” refers to a structure that is the lowest in terms of energy on the potential energy surface of a specific molecular system. It is routinely used in physical chemistry or cluster literature. In this work, we performed a DFT investigation on the structures, kinetic stability, bonding characters, and aromaticity of the CAl_4_X_4_ (X = Te, Po) cluster. It should be noted that CAl_4_X_4_ (X = Te, Po) are the first series of 40 valence-electron (VE) ptC species, which further broadens the electron counting rules for ptC clusters. The perfect CAl_4_X_4_ (X = Te, Po) molecular stars will further enrich the ptC family and provide new ideas for researchers to carry out relevant designs. To the best of our knowledge, there have been no theoretical or experimental investigations reported to date on the ptC CAl_4_X_4_ (X = Te, Po) clusters. 

## 2. Computational Details

The GM searches were performed for CAl_4_X_4_ (X = Te, Po) clusters using the Coalescence Kick (CK) approach [25,26,27] at the B3LYP/def2-SVP level [28,29], with 5000 structural points being probed for each species (including singlet and triplet). Subsequently, the low-lying isomers were further optimized at the B2PLYP-D3(BJ)/def2-TZVPP level [30,31,32]. The vibrational frequency calculations were calculated at the same level to confirm that the reported structures are true minima. In order to ensure energetic accuracy, single-point CCSD(T)/def2-TZVPP calculations were performed to benchmark energies for the top five low-lying isomers at the B2PLYP-D3(BJ) geometries [33,34]. The relative energies of isomers were determined by the CCSD(T)/def2-TZVPP energies plus the B2PLYP-D3(BJ)/def2-TZVPP zero-point energy corrections.

Natural bond orbital (NBO) analyses [35] were performed to obtain Wiberg bond indices (WBIs) and natural atomic charges. Born–Oppenheimer molecular dynamics (BOMD) [36] simulations were carried out at the B3LYP/def2-SVP level to characterize the dynamic stability. Canonical molecular orbitals (CMOs) and adaptive natural density partitioning (AdNDP) [37,38] analyses were performed to gain the chemical bonding nature of these peculiar ptC species. Nucleus-independent chemical shift (NICS) [39], and iso-chemical shielding surfaces in the z-direction (ICSS_zz_) [40] calculations were performed to assess π/σ aromaticity. All electronic structure calculations were performed using the Gaussian 16 package [41].

## 3. Result and Discussion

### 3.1. Designing the ptC CAl_4_X_4_ (X = Te, Po) Species

The current study is primarily motivated by the previously reported ppB BAl_5_$S_{5}^{+}$, ppC CAl_5_$O_{5}^{+}$, CB_5_$S_{5}^{+}$, and quasi-ptC CBe_4_Au_4_ clusters [21,22,23,42]. The hypothetical D_4h_ CAl_4_ is only an unstable third-order saddle point, which is energetically 17.7 kcal mol^−1^ higher than T_d_ CAl_4_ at the B2PLYP-D3(BJ)/def2-TZVPP level. How does the introduction of peripheral auxiliary atoms affect the structure of the central carbon atom in the T_d_ CAl_4_ cluster? Can we flatten CAl_4_ with fewer Te/Po atoms?

**Scheme 1:**
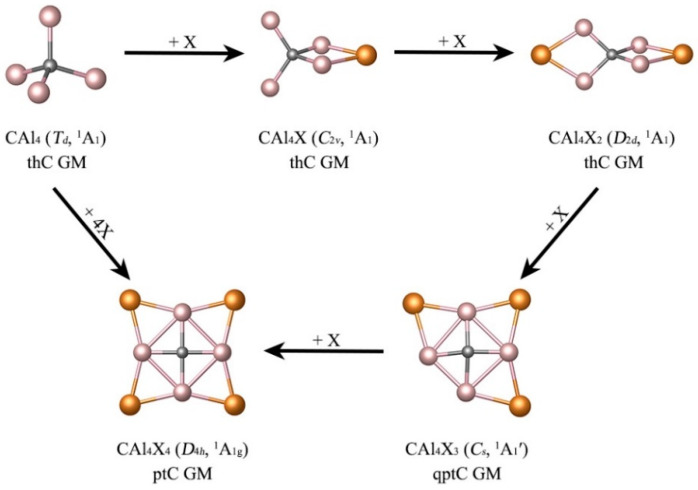
Flatten the tetrahedral structure of the CAl_4_ cluster by stepwise introducing the X (X = Te, Po) atoms.

Scheme 1 depictures the structure evolution of the CAl_4_ cluster by stepwise introducing the X (X = Te, Po) atoms. The calculated Al–Al distance in the T_d_ CAl_4_ cluster is 3.30 Å at the B2PLYP-D3(BJ)/def2-TZVPP level, which significantly exceeds the covalent bond length of Al–Al (2.52 Å), suggesting there is no chemical bonding between them. In order to know the minimum number of auxiliary atoms needed to obtain a planar structure, we made a series of attempts. We added an X (X = Te, Po) atom to the structure of CAl_4_ and the structure changed to C_2v_ symmetry. It should be noted that the newly introduced X atom has little effect on the bonding of the central carbon atom. After adding two x atoms, the thC structure was still maintained. As shown in Scheme 1, we cannot flatten it by adding only one or two X (X = Te, Po) bridging atoms to the Al–Al edges of T_d_ CAl_4_. When the third X atom is added, the tetrahedral structure of CAl_4_ changes radically. In other words, three peripheral X atoms can almost make the central structure of carbon in the system transition from thC to ptC. The C atoms are above 0.47 and 0.40 Å the Al_4_ planes in the CAl_4_X_3_ (X = Te, Po) species, respectively. Thus, CAl_4_X_3_ (X = Te, Po) can only be seen as the quasi-ptC (qptC) clusters. The addition of the fourth X atom is very critical because it can completely flatten the structure of T_d_ CAl_4_. Thus, the cumulative flattening effect of four X (X = Te, Po) atoms is strong enough to convert one thC into one ptC. In other words, introducing four peripheral Te/Po auxiliary atoms is an effective strategy to flatten the tetrahedral structure of CAl_4_. This may provide a new idea for us to design other planar hypercoordinate carbon systems.

### 3.2. Structures and Stability

**Figure 1:**
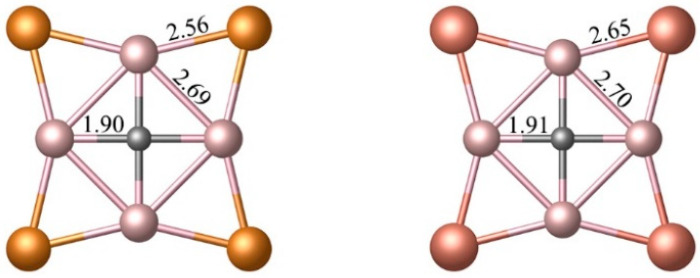
Optimized global minimum (GM) structures of the CAl_4_X_4_ (X = Te, Po) cluster at the B2PLYP-D3(BJ)/def2-TZVPP level. The bond distances are presented (in Å).

**Figure 2:**
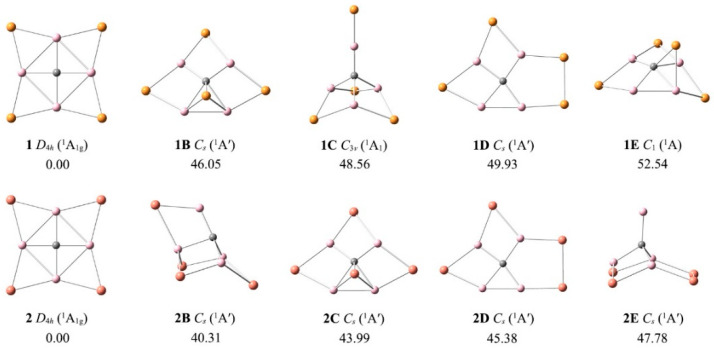
Optimized ptC global-minimum structures **1** and **2** of CAl_4_X_4_ (X = Te, Po) clusters and their four lowest-lying isomers (***n*B**–***n*E**) at the B2PLYP-D3(BJ)/def2-TZVPP level. The relative energies are listed in kcal mol^−1^ at the single-point CCSD(T)/def2-TZVPP//B2PLYP-D3(BJ)/def2-TZVPP level, with zero-point energy (ZPE) corrections at B2PLYP-D3(BJ).

As shown in Figure 1 and Figure 2, the GMs of CAl_4_X_4_ (X = Te, Po) possess the perfect quadrangular star structures, with the ptC atoms in the centers. *D*_4*h*_ CAl_4_Te_4_ (**1**) and CAl_4_Po_4_ (**2**) are the true GMs, with them being reasonably well separated from alternative structures by at least 46.05/40.31 kcal mol^−1^ at the single-point CCSD(T)/def2-TZVPP//B2PLYP-D3(BJ)/def2-TZVPP level. If one bridge X (X = Te, Po) atom in the GM structure moves upward to form a bond with the central carbon atom, its coordination changes from bridge di-coordination (µ^2^-X) to face-capping tri-coordination (µ^3^-X); then, isomer **1B**/**2C** can be obtained. It should be noted that the carbon atom in **1B**/**2C** forms a typical penta-coordination. The C atom in **2B** possesses irregular tetrahedral coordination, while all Po atoms remain in the bridge-based di-coordination mode. Among the isomers, there are the thC structures **1C** and **2E**, which are 48.56 and 47.78 kcal mol^−1^ higher than the GMs in energies at the CCSD(T) level, respectively. It should be noted that there is one terminal Al–Te group bonded to the central carbon atom in **1C**. If a bridge X (X = Te, Po) atom in the GM structure is translated into a neighboring bridge atom so that they are bonded to each other, the 1D/2D isomer can be formed. Interestingly, there is one peculiar Te–Te/Po–Po bond in the ptC **1D**/**2D**. When the µ^3^-Te atom in structure **1B** is slightly shifted to form a di-coordination (µ^2^-Te), the **1E** isomer can be obtained. The center C in **1E** is still a penta-coordinate atom. In terms of energy, **1** and **2** have absolute advantages over the other low-lying isomers. Note the *T_d_* CAl_4_X_4_ (X = Te, Po) are the local minima, which are 108.03 and 64.57 kcal mol^−1^ higher than the GMs at the CCSD(T)/def2-TZVPP//B2PLYP-D3(BJ)/def2-TZVPP level, respectively. Thus, the CAl_4_X_4_ (X = Te, Po) systems are unique in terms of potential landscapes.

**Figure 3:**
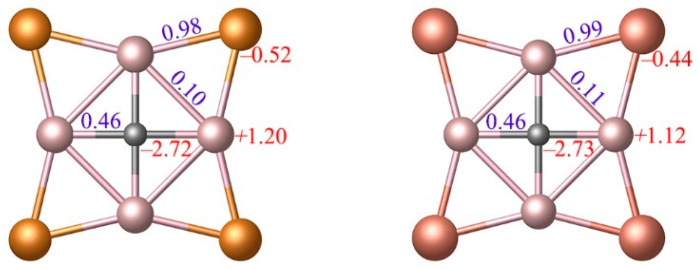
The Wiberg bond indices (WBIs, in the blue color) and atomic nature charges (q, |e|, in the red color) for **1** and **2** from the natural bond orbital (NBO) analyses.

As depictured in Figure 1 and Figure 3, the C–Al, Al–Al, and Al–Te distances in cluster **1** are 1.90, 2.69, and 2.56 Å, respectively. The C–Al bonding is of mixed covalent/ionic nature with the WBI_C–Al_ 0.46. The WBI_Al–Al_ (0.10) is relatively small, indicating that there is only weak covalent bonding between the Al ligands. The WBI_Al–Te_ (0.98) indicates that there is strong covalent bonding between the Al ligand and the Te auxiliary atom, which contributes to the planarity and rigidity of the overall structure. 

The difference in atomic electronegativity determines the charge distribution of the ptC **1** cluster. The electronegativity is 2.6/1.6/2.1 for C/Al/Te, respectively. As shown in Figure 3, the C center in **1** carries a natural charge of −2.72 |e|, whereas Te atoms are slightly negatively charged (−0.52 |e|) and Al positively charged (+1.20 |e|). There is considerable electron transfer from the Al ligands to the more electronegative C center, and the interactions between them exhibit clear ionic characteristics. From the perspective of electrostatic interaction, this negative-positive-negative charge distribution is conducive to the stability of the structure. The valence population of the central carbon in **1** is [He]2s^1.48^2$p_{x}^{1.79}$2$p_{y}^{1.79}$2$p_{z}^{1.65}$. Since the atomic radius of Po is larger than that of Te, the Al–Po (2.65 Å) bond distance in **2** is significantly longer than the Al–Te (2.56 Å) bond distance in **1**. However, the difference in peripheral atoms has little effect on the C–Al and Al–Al bond distances of the square CAl_4_ unit. As shown in Figure 3, the Wiberg bond indices (WBIs) and natural atomic charges in **2** are basically similar to those in **1**.

**Figure 4:**
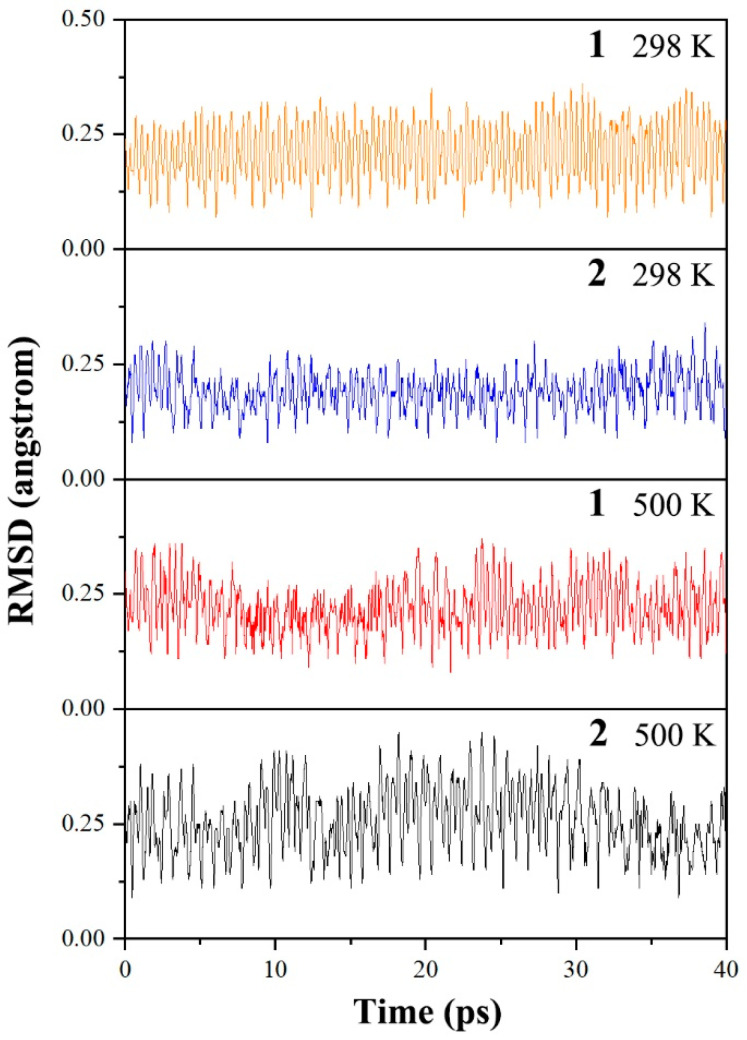
Calculated root-mean-square deviations (RMSDs) of GM clusters **1** and **2** of CAl_4_X_4_ (X = Te, Po) during the Born–Oppenheimer molecular dynamics (BOMD) simulations at 298 K and 500 K.

The HOMO–LUMO energy gap is another quantity that is frequently used to characterize the electronic stabilities of the target clusters. It indicates to some extent the ability of clusters to react to each other in chemical reactions. A larger value of the HOMO–LUMO gap usually indicates higher chemical stability. The ptC GM species, *D*_4*h*_ CAl_4_Te_4_ (**1**) and CAl_4_Po_4_ (**2**), have sizable HOMO–LUMO gaps (5.86 and 5.56 eV, respectively), suggesting that these neutral ptC species are electronically robust. From the point of view of experimental characterization, the dynamic stability of clusters is as important as the thermodynamic stability. To probe the dynamic stability, BOMD simulations were performed for **1** and **2** at the B3LYP/def2-SVP level for 40 ps at both 298 and 500 K. The kinetic stability of **1** and **2** can be evaluated by examining the structural evolution during the BOMD simulations, as quantified by the root-mean-square deviations (RMSDs) in Figure 4. The RMSDs of molecules in each frame are usually calculated with the first frame of the trajectory as the reference structure and the curve is drawn. The smaller the RMSD value fluctuation, the less easily the structure isomerizes. As shown in Figure 4, **1** and **2** are dynamically robust at 298 and 500 K, respectively, and maintain their ptC structures against isomerization and decomposition during the simulations. With the increase in temperature, the corresponding RMSD value increased. It should be noted that the major spikes in **1** and **2** are caused by the inversion vibration of ptC up and down the Al_4_ plane, suggesting that the Al_4_ ring is somewhat soft. The low WBI_Al–Al_ 0.10–0.11 further verified this understanding.

### 3.3. Chemical Bonding

**Figure 5:**
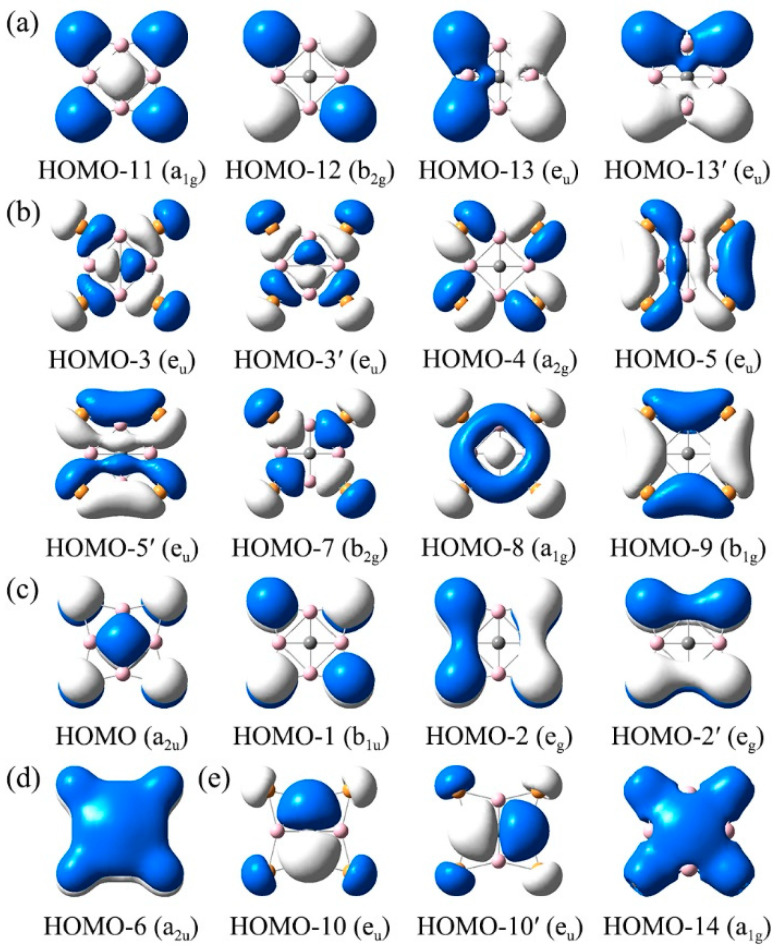
Analysis of canonical molecular orbitals (CMOs) of the *D*_4*h*_ CAl_4_Te_4_ (**1**) cluster. (**a**) Four lone pairs CMOs for one-center two-electron (1c-2e) of Te atoms. (**b**) Eight (2c-2e) Al-Te σ CMOs. (**c**) Four π CMOs for (3c-2e) Al-Te-Al bonds. (**d**) One delocalized π CMO over CAl_4_ unit. (**e**) Three delocalized σ CMOs over CAl_4_ unit.

Chemical bond analysis is important to help us understand the good thermodynamic and kinetic stability of the ptC CAl_4_X_4_ (X = Te, Po) clusters. Chemical bonding in the CAl_4_X_4_ (X = Te, Po) can be understood via CMO analysis, aided with orbital composition analysis (Tables S1 and S2). Note that the CMOs are fundamental in understanding the bonding nature in a molecular system. As shown in Figure 5, twenty occupied CMOs of *D*_4*h*_ CAl_4_X_4_ (**1**) can be divided into five subsystems. Subset (a) has four CMOs: HOMO-11, HOMO-12, and degenerated HOMO-13. This subset of CMOs is largely Te 5s based, which can be classified as four lone pairs (LPs) of four Te atoms. Subset (b) has eight CMOs, corresponding to localized two-center two-electron (2c-2e) Al-Te bonds. Subset (c) has three π-type CMOs (including HOMO, HOMO-1, and degenerate HOMO-2), corresponding to delocalized three-center two-electron (3c-2e) Al-Te-Al bonds. Subset (d) is the π framework on the CAl_4_ unit and involves only the HOMO-6. Lastly, subset (e) includes the degenerated HOMO-10 and HOMO-14, which are 6σ systems with major contributions from the CAl_4_ core. Thus, one π CMO (d) and three delocalized σ CMOs (e) suggest that there is two-fold delocalization in *D*_4*h*_ CAl_4_Te_4_ (**1**), that is, double (2π and 6σ) aromaticity.

**Figure 6:**
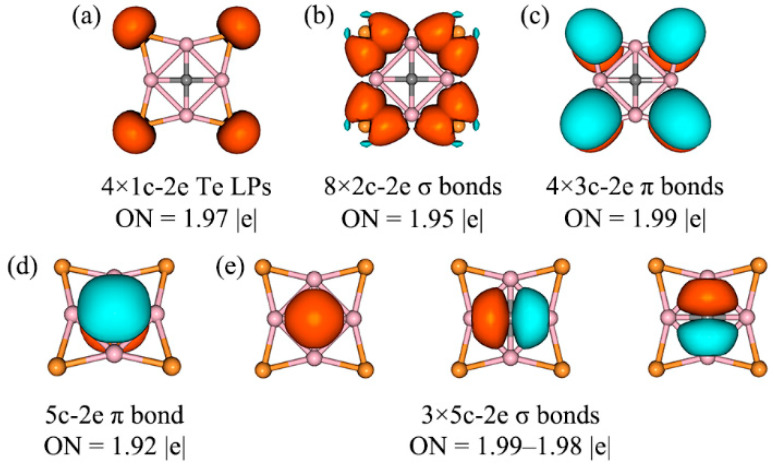
Chemical bonding pattern for the Cal_4_Te_4_ cluster, according to the adaptive natural density partitioning (AdNDP) analysis. Occupation numbers (Ons) are shown. (**a**) Four lone pairs of Te atoms. (**b**) Eight (2c-2e) Al-Te σ bonds. (**c**) Four 3c-2e Al-Te-Al π bonds. (**d**) One delocalized π bond over CAl_4_ unit. (**e**) Three delocalized σ bonds over CAl_4_ unit.

Compared with molecular orbital analysis, adaptive natural density partitioning (AdNDP) analysis is more intuitive. AdNDP is an extension of the NBO analysis, which is an efficient and visual approach to the interpretation of molecular orbital-based wave functions. AdNDP analyses recover typical Lewis’s bonding elements (LPs and 2c-2e bonds) and novel delocalized *n*c-2e (*n* ≥ 3) bonds. Boldyrev and coworkers found that the pattern of *n*c-2e remained qualitatively the same when they used different basis sets.^37^ In other words, the AdNDP analysis is insensitive to the theoretical method and basis sets. In the current work, AdNDP analyses were performed at the B3LYP/def2-TZVPP level using the Multiwfn program. AdNDP is sensitive to the occupation number (ON) threshold; the ideal ON value approaches 2. Therefore, researchers are responsible for the choice of the search strategy and acceptance of the final bonding pattern. The AdNDP scheme for CAl_4_Te_4_ is illustrated in Figure 6. CAl_4_Te_4_ (**1**) is a 40 VE system. There are four 1c-2e lone pairs (LPs) for four Te atoms, eight Al–Te 2c-2e σ bonds, and four Al–Te–Al 3c-2e π bonds on the periphery. Due to the polar bonding between Al and Te, the Al-Te-Al 3c-2e π bond(s) is mainly contributed by the LPs of Te atoms. If the Al–Te–Al 3c-2e π bond is approximated as a lone pair of Te, the ON is 1.76 |e|. There are obvious back-bonding characters in these Te→Al π bonds, which are similar to those 3c-2e B–S–B π bonds in the ppC CB_5_$S_{5}^{+}$ cluster. Regarding the bonding within the ptC CAl_4_ unit, there is one 5c-2e π bond and three 5c-2e σ bonds. Therefore, these four orbitals offer 2π and 6σ double aromaticity according to the Hückel 4*n* + 2 rule, which is beneficial to stabilize the ptC structure. It should be noted that the occupation numbers (ON) range from 1.92 to 1.99 |e|, which is reasonable in comparison with the expected value (2.00|e|). The CMOs and AdNDP bonding pattern of CAl_4_Po_4_ is similar to CAl_4_Te_4_, which is shown in Figures S1 and S2. 

### 3.4. Double Aromaticity

**Figure 7:**
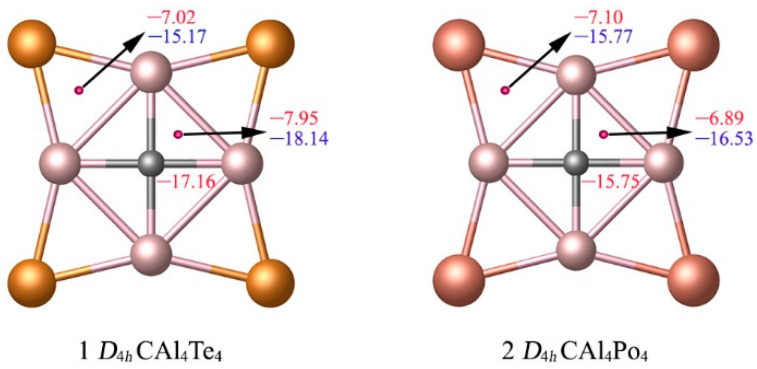
Nucleus-independent chemical shifts (NICSs) for clusters **1** and **2**. NICS (0), shown in blue, is calculated at the center of a triangle. NICS (1), shown in red, is calculated at 1 Å above the center of a triangle and above the C center.

The nucleus-independent chemical shift (NICS) calculations were performed to quantitatively characterize the aromaticity of the CAl_4_X_4_ (X = Te, Po) clusters. Since NICS is given chemical shielding at many points, aromaticity can therefore be evaluated in more detail. In addition, the center of the ring is conveniently defined, so NICS is more suitable for plane systems. The negative value of NICS(0)/NICS(1) is a semi-quantitative representation of σ/π aromaticity. As shown in Figure 7, the NICS(0) values at the geometric center of Al–Te–Al and Al–C–Al triangles are −15.17 ppm and −18.14 ppm, respectively, suggesting that CAl_4_Te_4_ possesses good σ aromaticity. Accordingly, the NICS(1) values of the Al–Te–Al and Al–C–Al triangles are −7.02 ppm and −7.95 ppm, respectively. In addition, the NICS(1) value of **1** at 1 Å above the central C atom is −17.16 ppm. The situation of CAl_4_Po_4_ (**2**) is similar to CAl_4_Te_4_ (**1**). Thus, **1** and **2** have both π and σ double aromaticity, which is consistent with the conclusion obtained from the AdNDP analysis.

**Figure 8:**
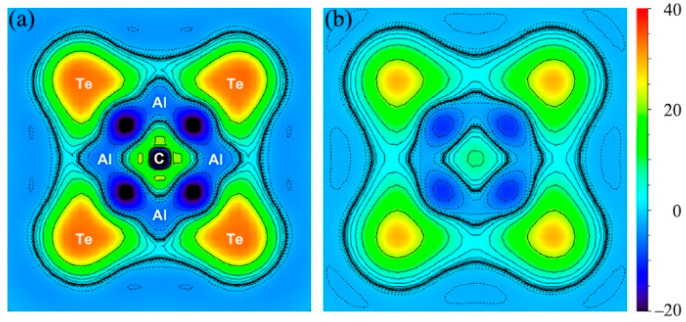
Color-filled maps of (**a**) ICSS(0)zz and (**b**) ICSS(1)zz (in ppm) for the CAl_4_Te_4_ (**1**) cluster. Positive values indicate aromaticity. The 0 and 1 in parentheses represent the height above the molecular planes (in Å).

However, it seems a little inadequate to reveal the aromaticity of the system through the NICS values of a few points. The magnetic criterion isochemical shielding surface (ICSS) calculation is handled in a three-dimensional grid of lattice points, and direction and anisotropy effects can be quantified in a more straightforward way. To more intuitively observe the aromaticity, the color-filled maps of ICSS_zz_(0) and ICSS_zz_ (1) are shown in Figure 8. Note here that positive ICSS_zz_ values indicate diatropic ring currents and aromaticity. Similarly, Figure 8a,b indicates that CAl_4_Te_4_ has σ and π double aromaticity. The situation is similar for CAl_4_Po_4_, as shown in Figure S3. Thus, the ICSS_zz_ results indicate that the ptC CAl_4_X_4_ (X = Te, Po) clusters possess σ and π double aromaticity, which is consistent with the conclusions obtained from the previous analysis of CMOs, AdNDP, and NICS.

### 3.5. Simulated IR Spectrums

**Figure 9:**
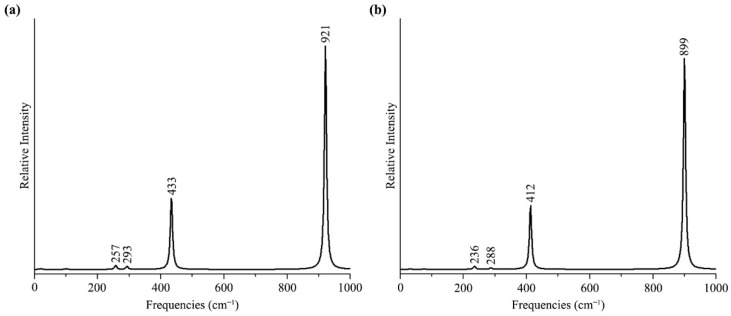
Simulated IR spectrums of CAl_4_Te_4_ (**a**) and CAl_4_Te_4_ (**b**) at the B2PLYP-D3(BJ)/def2-TZVPP level.

In order to facilitate future experimental characterization, the infrared (IR) spectrums of ptC stars **1** (D_4h_ CAl_4_Te_4_) and **2** (D_4h_ CAl_4_Po_4_) were simulated at the B2PLYP-D3(BJ)/def2-TZVPP level. As shown in Figure 9a, the strongest IR absorption peak occurs at 921 cm^−1^, which mainly originates from in-plane asymmetrical C–Al stretching vibrations. It should be noted that this vibration is accompanied by some contributions of C–Te vibration and has the characteristics of coupled vibration. The sub-strong peak at 433 cm^−1^ corresponds to asymmetrical Al–Te in-plane stretching vibrations. The weak peak at 293 cm^−1^ is the result of the up and down movements of the ptC center within the Al_4_ ring along the molecular C_4_ symmetric axis. The weak peak at 257 cm^−1^ is mainly generated by coupled Al–Te inplane bending vibrations. As shown in Figure 9b, the simulated infrared spectrum of **2** is basically similar to that of **1**, which will not be repeated here.

## 4. Conclusions

In conclusion, we have designed 40VE CAl_4_X_4_ (X = Te, Po) clusters with planar tetracoordinate carbon (ptC). The ptC CAl_4_X_4_ (X = Te, Po) clusters are well defined on the potential energy surface. Bonding analysis reveals 2π + 6σ aromaticity for these ptC clusters. Thus, the 40VE CAl_4_X_4_ (X = Te, Po) system features ptC geometry. The current findings suggest further opportunities to design novel planar hypercoordinate carbons via tuning ligands and auxiliary atoms and altering the electron counting.

## Data Availability

All data reported in this study are available upon request by contact with the corresponding author.

## Supplementary Materials

The following supporting information can be downloaded at: https://www.mdpi.com/article/10.3390/molecules28073280/s1, Figure S1: Analysis of canonical molecular orbitals (CMOs) of *D*_4*h*_ CAl_4_Po_4_ (**2**) cluster; Figure S2: Chemical bonding pattern for CAl_4_Po_4_ (**2**) cluster, according to the adaptive natural density partitioning (AdNDP) analysis. Occupation numbers (ONs) are shown; Figure S3: Color-filled maps of ICSSzz (in ppm) for the CAl_4_Po_4_ (**2**) cluster. Positive values indicate aromaticity. 0 and 1 in parentheses represent the height above the molecular planes (in Å).; Table S1: Orbital composition analysis of canonical molecular orbitals (CMOs) of the global-minimum structure **1** (*D*_4*h*_, ^1^A_1g_) of CAl_4_Te_4_ cluster.; Table S2: Orbital composition analysis of canonical molecular orbitals (CMOs) of the global-minimum structure **2** (*D*_4*h*_, ^1^A_1g_) of CAl_4_Po_4_ cluster.
