# Supplementary material for: CAl4X4 (X = Te, Po): Double Aromatic Molecular Stars Containing Planar Tetracoordinate Carbon Atoms
Source: Molecules. 2023 Apr 6;28(7):3280. doi: 10.3390/molecules28073280 (PMC10096394; doi:10.3390/molecules28073280)
Supplement: Supplementary file 1 [file molecules-28-03280-s001.zip › molecules-2324676-supplementary.pdf]

SUPPLEMENTARY INFORMATION

CAl_4X_4 (X = Te, Po): Double Aromatic Molecular Stars Containing Planar Tetracoordinate Carbon Atoms

Li-Xia Bai and Jin-Chang Guo *

Nanocluster Laboratory, Institute of Molecular Science, Shanxi University, Taiyuan 030006, China; bailixia2021@163.com

* Correspondence: guojc@sxu.edu.cn

Table of Contents

Table S1. Orbital composition analysis of canonical molecular orbitals (CMOs) of the global-minimum structure **1** (D_{4h}, $^1A_{1g}$) of CAl_4Te_4 cluster.

Table S2. Orbital composition analysis of canonical molecular orbitals (CMOs) of the global-minimum structure **2** (D_{4h}, $^1A_{1g}$) of CAl_4Po_4 cluster.

Figure S1. Analysis of canonical molecular orbitals (CMOs) of D_{4h} CAl_4Po_4 (**2**) cluster.

Figure S2. Chemical bonding pattern for CAl_4Po_4 (**2**) cluster, according to the adaptive natural density partitioning (AdNDP) analysis. Occupation numbers (ONs) are shown.

Figure S3. Color-filled maps of $ICSS_{zz}$ (in ppm) for the CAl_4Po_4 (**2**) cluster. Positive values indicate aromaticity. 0 and 1 in parentheses represent the height above the molecular planes (in Å).

Cartesian coordinates of optimized structures of the species reported in the text.

Table S1. Orbital composition analysis of canonical molecular orbitals (CMOs) of the global-minimum structure **1** (D_{4h}, $^{1}A_{1g}$) of CAl_4Te_4 cluster.

CMO	C (%)		Al_4 (%)		Te_4 (%)		MOs Energies (eV)
	s/p	total	s/p	total	s/p	total	
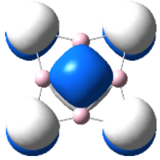 HOMO (a_{2u})	0.00/**38.13**	**38.13**	0.00/0.00	0.00	0.00/**61.09**	**61.09**	-6.94
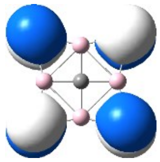 HOMO-1 (b_{1u})	0.00/0.00	0.00	0.00/0.00	0.00	0.00/**97.97**	**97.97**	-7.48
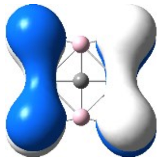 HOMO-2 (e_g)	0.00/0.00	0.00	0.00/13.94	13.94	0.00/**84.86**	**84.86**	-7.89
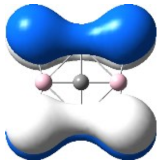 HOMO-2′(e_g)	0.00/0.00	0.00	0.00/13.94	13.94	0.00/**84.86**	**84.86**	-7.89
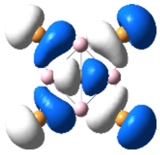 HOMO-3 (e_u)	0.00/**24.28**	**24.28**	0.00/4.65	4.65	0.00/**67.48**	**67.48**	-7.97
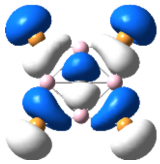 HOMO-3′(e_u)	0.00/**24.28**	**24.28**	0.00/4.65	4.65	0.00/**67.48**	**67.48**	-7.97

CMO	C (%)		Al_4 (%)		Te_4 (%)		MOs Energies (eV)
	s/p	total	s/p	total	s/p	total	
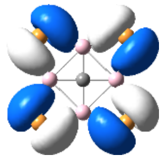 HOMO-4 (a_{2g})	0.00/0.00	0.00	0.00/13.72	13.72	0.00/**85.13**	**85.13**	-8.13
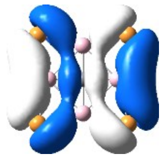 HOMO-5 (e_u)	0.00/8.31	8.31	11.93/**16.46**	**28.39**	0.00/**61.84**	**61.84**	-9.23
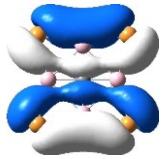 HOMO-5′ (e_u)	0.00/8.31	8.31	11.93/**16.46**	**28.39**	0.00/**61.84**	**61.84**	-9.23
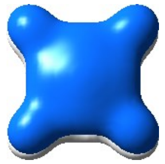 HOMO-6 (a_{2u})	0.00/**44.32**	**44.32**	0.00/**28.34**	**28.34**	0.00/**26.13**	**26.13**	-9.37
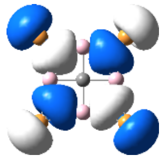 HOMO-7 (b_{2g})	0.00/0.00	0.00	0.00/14.64	14.64	7.96/**75.40**	**83.36**	-9.56
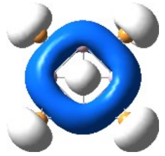 HOMO-8 (a_{1g})	8.00/0.00	8.00	14.59/0.00	14.59	8.92/**65.66**	**74.58**	-10.13
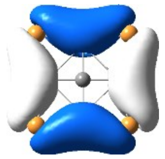 HOMO-9 (b_{1g})	0.00/0.00	0.00	**31.97**/12.82	**44.79**	0.00/**53.30**	**53.30**	-10.67

Table S2. Orbital composition analysis of canonical molecular orbitals (CMOs) of the global-minimum structure **2** (D_{4h}, $^1A_{1g}$) of CAl_4Po_4 cluster.

CMO	C (%)		Al_4 (%)		Po_4 (%)		MOs Energies (eV)
	s/p	total	s/p	total	s/p	total	
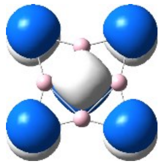 HOMO (a_{2u})	0.00/**32.98**	**32.98**	0.00/0.00	0.00	0.00/**65.90**	**65.90**	-6.69
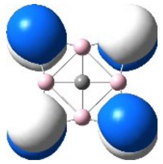 HOMO-1 (b_{1u})	0.00/0.00	0.00	0.00/0.00	0.00	0.00/**98.09**	**98.09**	-7.06
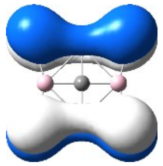 HOMO-2 (e_g)	0.00/0.00	0.00	0.00/14.32	14.32	0.00/**84.60**	**84.60**	-7.47
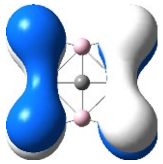 HOMO-2′(e_g)	0.00/0.00	0.00	0.00/14.32	14.32	0.00/**84.60**	**84.60**	-7.47
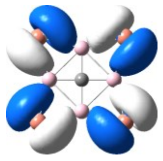 HOMO-3 (a_{2g})	0.00/0.00	0.00	0.00/13.20	13.20	0.00/**85.81**	**85.81**	-7.58
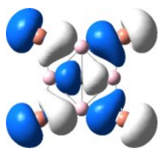 HOMO-4 (e_u)	0.00/**20.30**	**20.30**	0.00/7.24	7.24	0.00/**69.68**	**69.68**	-7.69

CMO	C (%)		Al_4 (%)		Po_4 (%)		MOs Energies (eV)
	s/p	total	s/p	total	s/p	total	
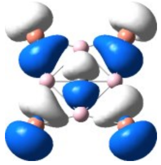 HOMO-4′ (e_u)	0.00/**20.30**	**20.30**	0.00/7.24	7.24	0.00/**69.68**	**69.68**	-7.69
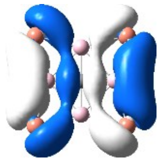 HOMO-5 (e_u)	0.00/11.76	11.76	13.18/**16.06**	**29.24**	0.00/**57.62**	**57.62**	-8.80
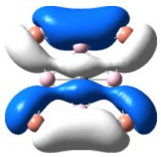 HOMO-5′ (e_u)	0.00/11.76	11.76	13.18/**16.06**	**29.24**	0.00/**57.62**	**57.62**	-8.80
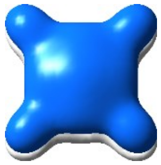 HOMO-6 (a_{2u})	0.00/**49.42**	**49.42**	0.00/**28.26**	**28.26**	0.00/**21.10**	**21.10**	-9.10
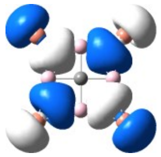 HOMO-7 (b_{2g})	0.00/0.00	0.00	0.00/**19.70**	**19.70**	6.03/**72.43**	**78.46**	-9.30
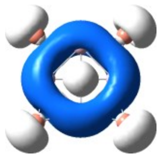 HOMO-8 (a_{1g})	8.88/0.00	8.88	**20.09**/0.00	**20.09**	6.44/**61.21**	**67.65**	-10.12
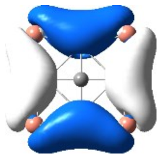 HOMO-9 (b_{1g})	0.00/0.00	0.00	**33.41**/13.76	**47.17**	0.00/**51.05**	**51.05**	-10.22

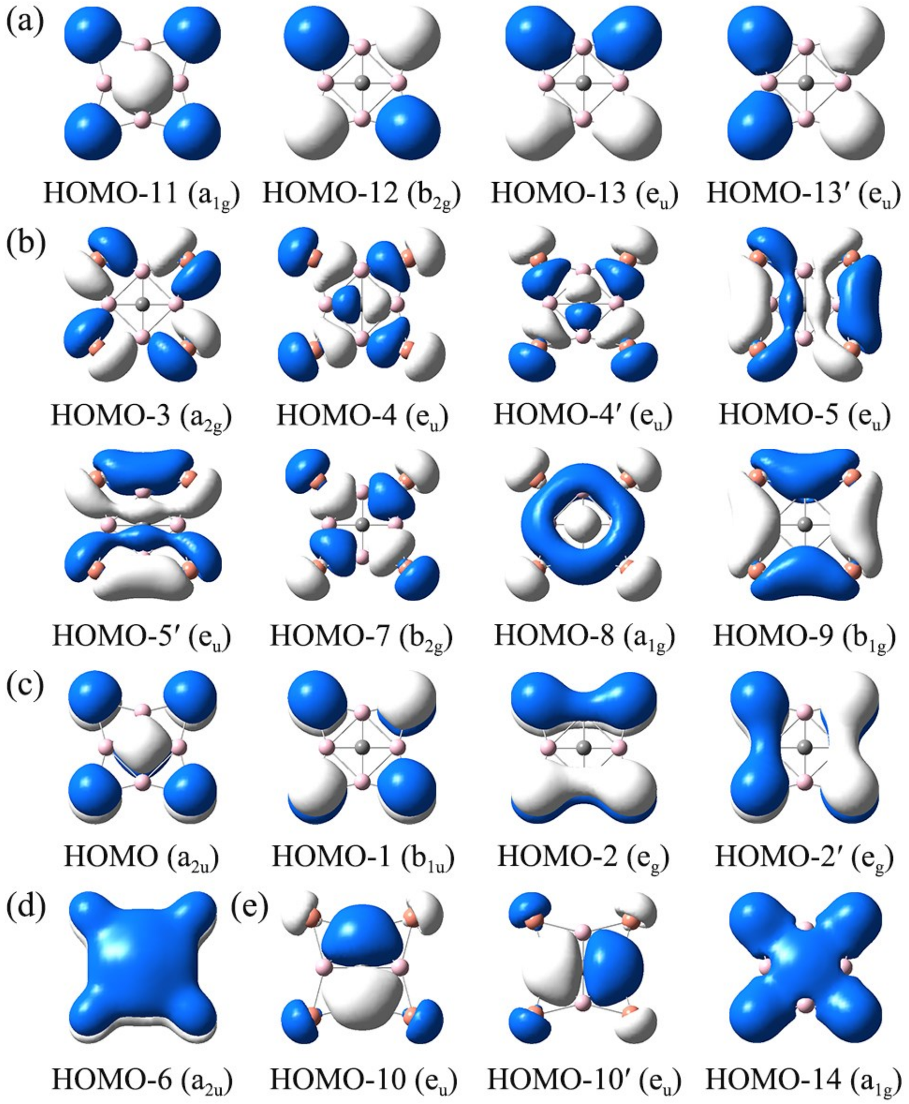

Figure S1. Analysis of canonical molecular orbitals (CMOs) of D_{4h} CAl_4Po_4 (**2**) cluster.

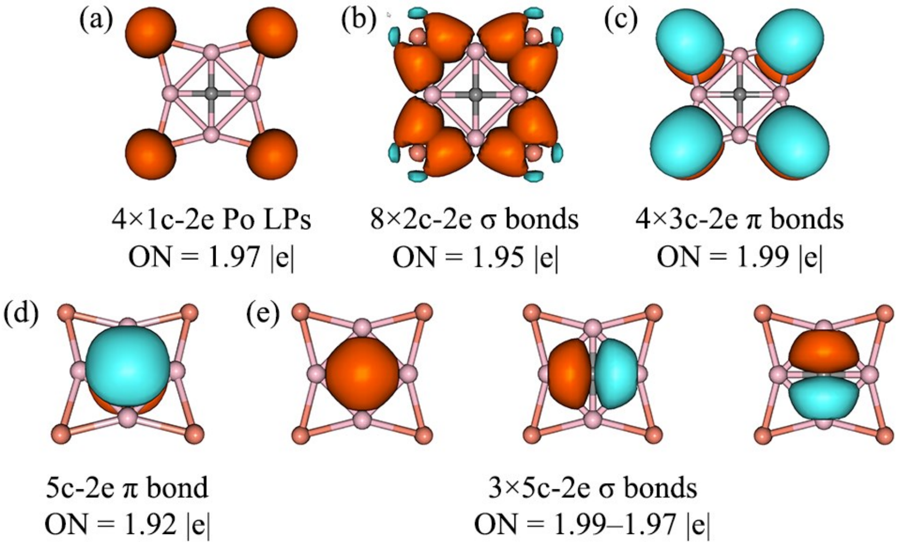

Figure S2. Chemical bonding pattern for CAl_4Po_4 (**2**) cluster, according to the adaptive natural density partitioning (AdNDP) analysis. Occupation numbers (ONs) are shown.

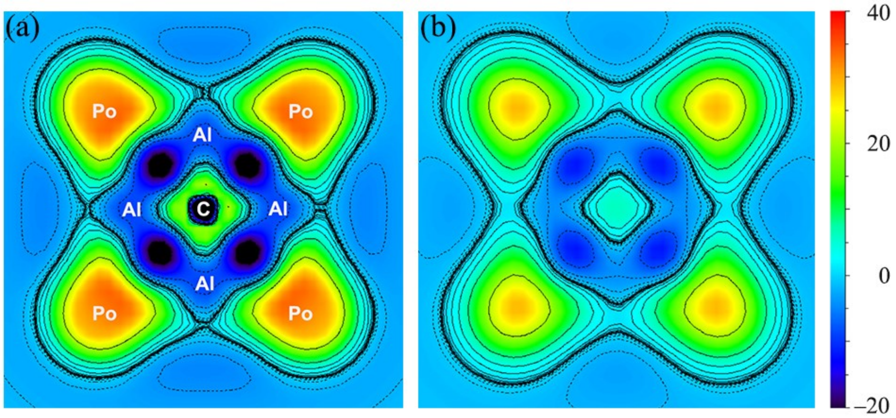

Figure S3. Color-filled maps of $ICSS_{zz}$ (in ppm) for the CAl_4Po_4 (**2**) cluster. Positive values indicate aromaticity. 0 and 1 in parentheses represent the height above the molecular planes (in Å).

Cartesian coordinates of optimized structures of the species reported in the text.

1

Al	0.00000000	1.90473200	0.00000000
Al	1.90473200	0.00000000	0.00000000
Al	-1.90473200	0.00000000	0.00000000
Al	0.00000000	-1.90473200	0.00000000
C	0.00000000	0.00000000	0.00000000
Te	-2.49474500	2.49474500	0.00000000
Te	-2.49474500	-2.49474500	0.00000000
Te	2.49474500	2.49474500	0.00000000
Te	2.49474500	-2.49474500	0.00000000

1B

C	0.54429900	-0.04779300	0.00000000
Al	-0.42538700	0.88076000	1.39335700
Al	-0.42538700	0.88076000	-1.39335700
Al	0.45302200	-1.76989500	1.21302600
Al	0.45302200	-1.76989500	-1.21302600
Te	-0.42538700	-0.66169700	3.39065100
Te	2.63326700	-0.70184500	0.00000000
Te	-1.85911400	2.47532000	0.00000000
Te	-0.42538700	-0.66169700	-3.39065100

1C

C	0.00000000	0.00007500	0.55192100
Al	0.00000000	1.57152000	-0.60940500
Al	-1.36096600	-0.78566400	-0.60938800
Al	1.36096600	-0.78566400	-0.60938800
Al	0.00000000	0.00005100	2.42125400
Te	0.00000000	-2.70433200	-1.65605300
Te	0.00000000	-0.00034700	4.76095500
Te	-2.34200000	1.35218000	-1.65665300
Te	2.34200000	1.35218000	-1.65665300

1D

C	0.00000000	0.82757900	0.00000000
Al	0.12459800	2.81169400	0.00000000
Al	1.86483900	1.18012700	0.00000000
Al	-1.92228700	1.05004900	0.00000000
Al	-0.28766500	-1.05448000	0.00000000
Te	3.58804000	-0.66879100	0.00000000
Te	1.66282000	-2.67245000	0.00000000
Te	-2.34161800	3.55423900	0.00000000

Te	-2.85411400	-1.30533600	0.00000000

1E

C	-0.13989300	-0.68163100	0.48970800
Al	1.33059200	-1.88915000	-0.48063000
Al	1.09853400	0.83087900	0.08432000
Al	-1.60800200	0.55940800	0.39928800
Al	-1.13866300	-2.11256800	-0.42224100
Te	2.66283200	-0.06139500	-1.73934700
Te	-3.40713500	-0.92327800	-0.65792500
Te	1.35056800	-1.12447300	2.05444400
Te	-0.51073900	2.84065300	0.39114000

2

Al	0.00000000	1.91257100	0.00000000
Al	1.91257100	0.00000000	0.00000000
Al	-1.91257100	0.00000000	0.00000000
Al	0.00000000	-1.91257100	0.00000000
C	0.00000000	0.00000000	0.00000000
Po	-2.56453700	2.56453700	0.00000000
Po	-2.56453700	-2.56453700	0.00000000

Po	2.56453700	2.56453700	0.00000000
Po	2.56453700	-2.56453700	0.00000000

2B

C	-0.75347800	1.28561400	0.00000000
Al	0.63385000	1.42571100	1.35921900
Al	-2.56351100	1.00717200	0.00000000
Al	0.63385000	1.42571100	-1.35921900
Al	-0.62596500	-0.82001800	0.00000000
Po	0.63385000	-0.97460900	2.40781600
Po	-3.40826800	-1.37347700	0.00000000
Po	2.49180600	2.76061000	0.00000000
Po	0.63385000	-0.97460900	-2.40781600

2C

C	0.44801600	-0.18275100	0.00000000
Al	0.43630600	-1.87390000	1.21711600
Al	-0.44205300	0.81090400	-1.40287500
Al	-0.44205300	0.81090400	1.40287500
Al	0.43630600	-1.87390000	-1.21711600
Po	2.68955500	-0.71811000	0.00000000

Po	-0.44205300	-0.75264000	-3.49288300
Po	-0.44205300	-0.75264000	3.49288300
Po	-1.83567100	2.56546600	0.00000000

2D

C	0.00000000	0.92614000	0.00000000
Al	-0.30569500	-0.95811200	0.00000000
Al	-1.92426100	1.17509500	0.00000000
Al	0.15595100	2.91841800	0.00000000
Al	1.87325600	1.26412800	0.00000000
Po	-2.36332100	3.76484700	0.00000000
Po	-2.96006900	-1.22656700	0.00000000
Po	3.70514100	-0.58810900	0.00000000
Po	1.64931700	-2.69720300	0.00000000

2E

C	-1.39441700	1.13129300	0.00000000
Al	-0.64633100	0.20564100	1.51915400
Al	0.16363900	2.31587700	0.00000000
Al	-0.64633100	0.20564100	-1.51915400
Al	-3.25306800	1.72759800	0.00000000

Po	1.03522200	2.03678900	2.48150900
Po	1.03522200	2.03678900	-2.48150900
Po	-0.64633100	-2.42190600	1.48657900
Po	-0.64633100	-2.42190600	-1.48657900
